# Design and Optimization of Cationic Nanocapsules for Topical Delivery of Tretinoin: Application of the Box-Behnken Design, *In Vitro* Evaluation, and *Ex Vivo* Skin Deposition Study

**DOI:** 10.1155/2021/4603545

**Published:** 2021-12-12

**Authors:** Saeed Ebrahimi, Reza Mahjub, Rasool Haddadi, Seyed Yaser Vafaei

**Affiliations:** ^1^Department of Pharmaceutics, School of Pharmacy, Hamadan University of Medical Sciences, Hamadan, Iran; ^2^Medicinal Plants and Natural Products Research Center, Hamadan University of Medical Sciences, Hamadan, Iran; ^3^Department of Toxicology and Pharmacology, School of Pharmacy, Hamadan University of Medical Sciences, Hamadan, Iran

## Abstract

Cationic nanocapsules represent a promising approach for topical delivery purposes. We elaborated on a novel formulation based on the cationic nanocapsules to enhance the pharmacodynamic efficacy, user compliance, and photostability of tretinoin (TTN). To achieve this goal, TTN nanocapsules were prepared by the nanoprecipitation method. In order to statistically optimize formulation variables, a Box-Behnken design, using Design-Expert software, was employed. Three independent variables were evaluated: total weight of the cationic acrylic polymer (*X*_1_), oil volume (*X*_2_), and TTN amount (*X*_3_). The particle size and encapsulation efficiency percent (EE%) were selected as dependent variables. The optimal formulation demonstrated spherical morphology under scanning electron microscopy (SEM), optimum particle size of 116.3 nm, and high EE% of 83.2%. TTN-loaded nanocapsules improved photostability compared to its methanolic solution. The *in vitro* release study data showed that tretinoin was released in a sustained manner compared to the free drug. The *ex vivo* skin permeation study demonstrated that greater drug deposition into the epidermal region rather than the deep skin was observed with a gel containing TTN-loaded nanocapsules than that of drug solution, respectively. The skin irritation test revealed that the nanoencapsulation of the drug decreased its irritancy compared to the free drug. These results revealed the promising potential of cationic nanocapsules for topical delivery of tretinoin

## 1. Introduction

Tretinoin (TTN) is a first-generation retinoid with keratolytic and anti-inflammatory activities which is used topically in the treatment of various dermatological diseases such as photoaging, acne, and psoriasis [1–3]. However, its use is strongly limited due to several disadvantages such as poor aqueous solubility, physicochemical instability, and topical adverse effects such as skin irritation, dryness, and scaling [4, 5].

Encapsulation of tretinoin with various delivery systems such as solid lipid nanoparticles [6, 7], liposomes [8], and niosomes [9, 10] has been proposed to help improve its stability, solubility, and efficiency. However, most reported systems showed only a limited improvement in the tretinoin loading ability and efficacy [11]. Therefore, the development of a carrier system with better efficacy and fewer side effects for tretinoin is still needed.

Polymeric nanoparticles have been considered promising carriers for cutaneous use [12]. The main advantages of these colloidal suspensions are improvement in the protection of the loaded active ingredient against physicochemical degradation as well as controlled release of drugs for a homogenous release [13, 14]. Nanocapsules are specific polymeric nanoparticles with a core-shell organization in which the polymeric wall surrounds an oily core [15]. These nanocapsules have important advantages, such as higher drug loading capacity and improved ability to protect the encapsulated drug from degradation [15, 16]. Moreover, they exhibit slow and sustained release of incorporated drugs which makes them suitable for cutaneous applications [17].

Among the polymers which are employed in the preparation of nanocapsules, the poly(ethyl acrylate, methyl-methacrylate) copolymer containing quaternary ammonium groups (Eudragit RS100) yields great interest due to its cationic charge [12]. It seems that cationic charge drug carriers adhere more strongly to negatively charged tissues and cell surfaces, which makes them suitable for topical formulations with prolonged residence time on the skin [18] and decreasing skin irritation of incorporated drugs [18].

Generally, dermal application of aqueous nanocapsules onto the skin is difficult due to their low viscosity and they are easily removed. So, in order to improve their rheological properties, spreadability, and skin residence time, nanocapsules are incorporated into the semisolid vehicles [19, 20].

The aim of this study was to develop topical TTN-loaded cationic nanocapsules to provide a slow release of drugs and higher permeability in the skin layers and enhance user compliance and photostability. Hence, optimal production parameters with optimum particle size and encapsulation efficiency were adjusted using the Box-Behnken statistical design. Moreover, the *ex vivo* skin permeation study of TTN from the optimum formulation was compared with the TTN solution. Furthermore the draize skin irritation test was performed to assess the irritation potential of TTN-loaded nanocapsules when embedded in a Carbopol gel on the rat skin.

## 2. Material and Methods

### 2.1. Chemicals

Tretinoin (TTN) was purchased from Olon S.p.A. (Italy). Eudragit RS100 was obtained from Degussa (Darmstadt, Germany). Sorbitan monooleate (Span 80), polysorbate 80 (Tween 80), and caprylic/capric triglyceride mixture were supplied from KLK OLEO (Malaysia), and Carbomer 940 (Carbopol® 940, Lubrizol, USA) and triethanolamine were obtained from Fluka Chemical (Switzerland). HPLC grade acetonitrile was acquired from Duksan (South Korea). And all chemicals and solvents presented pharmaceutical or HPLC grades.

### 2.2. Experimental Design

A three-level three-factor Box-Behnken (BB) design was employed to statistically optimize the formulation variables for preparing TTN-loaded lipid-core nanocapsules (LCNC) in order to obtain high EE% and optimum particle size. Development and evaluation of the experimental design were performed using Design-Expert® software (Version 7, Stat-Ease Inc., Minneapolis, MN, USA). A total of 15 experiments were run, 12 of which represent the midpoint of each edge of the multidimensional cube, and the remaining three are replicates of the cube's center point. Three independent variables were evaluated: total weight of the cationic acrylic polymer (*X*_1_), TTN amount (*X*_2_), and oil volume (*X*_3_). The encapsulation efficiency percent (EE%,*Y*_1_) and particle size (PS,*Y*_2_) were selected as the dependent variables. The independent variables (high, medium, and low levels) and dependent variables are reported in Table 1. The formations of the prepared TTN-LCNC are reported in Table 2. Desirability was calculated for the selection of the optimized formulation [21].

### 2.3. Preparation of TTN-LCNC

The nanocapsule suspensions were prepared by the interfacial deposition of the preformed polymer method [22, 23]. An organic phase composed of Eudragit® RS100 (0.125 g), which composes the particle shell, and the oily component of the particle core, capric/caprylic triglycerides (MCT) oil (0.200 g), Span 80® (0.076 g), and TTN (12.5 mg) were dissolved in 27 ml of acetone. After the solubilization of all components, the acetone solution was added dropwise to the aqueous phase (76 mg Tween 80® dissolved in 53 ml of deionized water) under moderate stirring for 30 min. Afterwards, acetone and part of the water were eliminated by evaporation at 40°C under reduced pressure to achieve a final volume of 25 ml. All preparations were protected from the light and kept in the dark [24].

### 2.4. Characterization of the NCs

#### 2.4.1. Particle Size and Zeta Potential

Particle sizes and zeta potentials (*n* = 3) were measured by photon correlation spectroscopy (Zetasizer Nanoseries, Malvern Instruments, ZEN3600, UK) after adequate dilution of an aliquot of the formulation in purified water at 25°C [25].

#### 2.4.2. Drug Content and Encapsulation Efficiency (EE%)

The total content of TTN in nanocapsule suspensions (*n* = 3) was assayed by diluting an aliquot of the sample in 25 ml acetone and submitting it to sonication for 30 min to extract the drug. Before injecting them into the HPLC system, the samples were filtered in a 0.45 *μ*m membrane. To determine encapsulation efficiency, an aliquot of the samples was placed in a 10,000 MW centrifugal filter device (Amicon® Ultra, Millipore) and the free drug was separated from the nanocapsules using the ultrafiltration/centrifugation technique at 8000 × g for 15 min. The EE% was calculated as the difference between the total and free concentrations of TTN, determined in the nanostructures and ultrafiltrate, respectively. TTN was quantified by High-Performance Liquid Chromatography (HPLC) on the Shimadzu system with a Shimadzu multiwavelength UV-VIS detector (SPD-20AV). The analysis was carried out at 25°C on a C18 column (4.6 mm 250 mm i.d., PerfectSil MZ). The mobile phase consisted of acetonitrile, Milli-Q water, and glacial acetic acid (85 : 14 : 1), filtered through a 0.45 *μ*m membrane filter (Millipore, USA) in the isocratic mode. The flow rate was 1.5 ml/min, and the detector was set at 355 nm

(1) $$\begin{array}{c} \text{EE}\%=\frac{\text{Total drug content}-\text{Free drug}}{\text{Total drug content}}\times 100, \end{array}$$

(2) $$\begin{array}{c} \%\text{Drug content}=\frac{\text{Total drug content}-\text{Free drug}}{\text{Nanoparticle weight}}\times 100. \end{array}$$

#### 2.4.3. Formulation Optimization

The optimized formulation was obtained using the Design-Expert® software on EE% to reach the maximum value and on particle size to obtain the minimum value (Table 1). The suggested optimized formulation was then developed and evaluated to check the validity of the optimal formulation factors (*n* = 3) and predicted responses given by the software [21].

#### 2.4.4. Photostability Studies

To evaluate the stability of TTN-loaded NCs, it was exposed to an ultraviolet (UV) lamp (long-wave UV light 366 nm, 2 light tubes 8 W each, CAMAG UV Cabinet, CAMAG Manufacturer, Switzerland). The 2 ml TTN-LCNC and 2 ml TTN methanolic solution (TTN-M-S) (in a 1 cm quartz cuvette) were exposed to UV radiation for 2 hours (h) at a fixed distance of 10 cm (*n* = 3). 500 *μ*l of the samples was withdrawn at 0, 10, 30, 50, 80, 100, and 120 min. Then, the samples were extracted using a centrifugal filter device as discussed previously and the amount of the drug was assayed by HPLC. Also, as a control, the TTN-LCNC and TTN-M-S coated with aluminum foil (UV protection) were evaluated in the same way [25].

#### 2.4.5. *In Vitro* Tretinoin Release Study

Release profiles of TTN were obtained by the dialysis diffusion technique at 37°C in 500 ml phosphate buffer (pH 7.4) with 49% of 2-propanol and 1% Tween 80® to keep the sink conditions. The samples, either tretinoin methanolic solution (TTN-M-S) or TTN-loaded nanocapsules, were placed in the dialysis bag (MWCO 12,000, Scientific Laboratory). This system was kept under continuous magnetic stirring of 150 rpm. Aliquots of 1 ml were withdrawn at predetermined periods and replaced by the same volume of the fresh medium. The amount of tretinoin was assessed using HPLC. The experiment was conducted in triplicate.

#### 2.4.6. Morphological Study

The appearance of the particles was evaluated using a scanning electron microscope (SEM) (Quanta 450, FEI Company, USA) [6]. First, a small amount of NC suspension was applied in a thin layer on an aluminum surface. Then, a thin layer of gold was applied to the particles using a sputter coater. Then, for SEM photography, the coated sample was placed in the main compartment of the instrument and examined with a voltage of 30.00 kV.

#### 2.4.7. Physicochemical Stability

The NC suspensions were stored for 3 months at 25°C and 4°C, and all samples were protected from the light and kept in the dark all the time. The samples were withdrawn at 0, 30, 60, and 90 days and were assayed by HPLC. Also, particle sizes and PDI and zeta potentials were measured by photon correlation spectroscopy (Zetasizer Nanoseries, Malvern Instruments, ZEN3600, UK) after adequate dilution with purified water. The analysis was performed at 25°C [26].

### 2.5. Preparation of Hydrogels Containing TTN-Loaded Nanocapsules (HG-TTN-LCNC)

Hydrogels (HGs) were prepared using mortar and pestle by adding nanocapsule suspensions (10 ml) in 0.7-gram (g) Carbomer 934 (HG-TTN-LCNC) [7]. For the HG preparation containing free TTN, they were solubilized in propylene glycol (1 ml) and incorporated into a HG previously prepared with distilled water and carbomer. The HGs obtained were named HG-TTN (containing non-nanoencapsulated TTN). The vehicle was prepared following the same methodology, dispersing 0.07 g Carbopol 934 in water (9 ml) and propylene glycol (1 ml). The percentage of the tretinoin in the gel was 0.05% *w*/*w*[7, 24].

### 2.6. Characterization of HG-TTN-LCNC

The pH values of the hydrogels were determined to be suitable for applied hydrogels onto the dermal region, using a calibrated pH meter (Sartorius, PB-11), after dilution of 1 g of the samples in 250 ml water (4% *w*/*v*) [7]. The rheological characteristics of the hydrogels were determined using a rotational viscometer (DVII Digital Viscometer, Brookfield Instruments, UK) and spindle SC34. The analysis was carried out at 25 ± 1°C[7, 24]. The total TTN content in the hydrogel formulation was assayed by diluting a sample aliquot in methanol and subjecting it to sonication for 15 min and centrifuging at 500 × g for 10 min. Samples were filtered through a 0.45 *μ*m membrane and injected into the HPLC system [24].

### 2.7. Animals

Male Wistar rats weighing 240–250 g were used in all experiments. The animals were kept in a temperature-controlled room (25 ± 2°C) on a 12 h light-dark cycle and supplied with standard diet and water. The animals were habituated to the experimental room for 3 days before the tests. All of the experiments were done in accordance with the National Institutes of Health Ethical Guidelines for the Care and Use of Laboratory Animals and approved by the Ethics Committee of Hamadan University of Medical Sciences with the ethical code of IR.UMSHA.REC.1397.255. Animals were randomly designated in different treatment groups, and all experiments were blindly performed.

### 2.8. *Ex Vivo* Skin Penetration Study

The study was conducted at 37°C using a Franz diffusion cell with a 15.0 ml capacity receptor compartment and a 2.54 cm^2^ diffusion area [7]. The abdominal rat skin was prepared using the modified technique (Joshi, Kaur et al. 2018). The receptor medium (50% phosphate buffer (pH = 7.4), 49% 2-propanol, and 1% Tween 80®) was constantly stirred during the experiment. The experiment was initiated by applying the encapsulated TTN gel (HG-TTN-LCNC), TTN-loaded gel (HG-TTN), and commercial gel (HG-C), each containing TTN equivalent to 0.3 mg into the donor compartment, directly onto the mounted skin. At 2, 4, 6, 8, 12, 16, 20, and 24 h later, 200 *μ*l of the receptor fluid was withdrawn and replaced with a fresh receptor medium. The concentration of TTN in each aliquot of the withdrawn receptor fluid was determined by HPLC as previously described [24].

### 2.9. *Ex Vivo* Skin Deposition Study

After completion of the *ex vivo* skin penetration study (after 24 hours), the rat skin mounted on the Franz diffusion cell was carefully taken off. The sample on the skin surface was carefully washed. Then, the cleaned rat skin tissue was washed thrice with Milli-Q water and dried on a lint-free cotton swab. The epidermis was separated from the dermis by means of heat application [27]. The separated skin samples were chopped into small pieces and placed in a flask with 5 ml methanol. The samples were mixed using a Vortex Mixer (Heidolph, REAX Top) for 120 seconds and then homogenized by an ULTRA-TURRAX® homogenizer (IKA® T10 B) for 45 minutes. Then, the samples were centrifuged at 8000 rpm for 15 minutes and the accumulated amount of TTN in the epidermis and dermis was extracted. After filtering the samples through a PTFE syringe filter (0.45 *μ*m), the filtrate sample was assayed using the validated HPLC technique [28, 29].

### 2.10. Skin Irritation Test

In our study, skin irritation tests were performed in two healthy white rabbits (each 3-4 kg). The animal procedures were performed according to the written approval of the Ethics Committee, Deputy of Research and Technology, Hamadan University of Medical Sciences (Approval ID: IR.UMSHA.REC.1397.255). The skin on both sides of bodies was shaved, and 4 points were marked on each side. 500 mg of all hydrogels containing tretinoin-loaded lipid-core nanocapsules (TTN-LCNC), tretinoin methanolic solution (TTN-M-S), blank lipid-core nanocapsules (HG-B-LCNC), and commercial gel was applied to the shaved surface of each rabbit, at a dose of 0.05% (*w*/*w*) tretinoin on 4 skin surface points with the area of 4 cm^2^. After 24 h, the parafilm adhered on the skin surface was taken off and the skin surface points were observed for any visible change such as erythema (redness, inflammation, and swelling) after 24, 48, and 72 h. The erythemal scores were reported from 0 to 4 according to Draize, where 0 means no erythema, 1 slight erythema, 2 moderate erythema, 3 moderate to severe erythema, and 4 severe erythema [30].

### 2.11. Statistical Analysis

All samples were prepared and analyzed at least in triplicate. Results are expressed as mean ± SD (standard deviation). The Box-Behnken response surface design and model fitting were accomplished by one-way analysis of variance (ANOVA) using Design-Expert® software (V.7.0.0). In this study, the comparison of two groups of data was performed using the two-sample independent *t*-test while the comparison between three or more groups was accomplished using ANOVA which was followed by the Tukey post hoc test. The significance level was set as 0.05 in all cases.

## 3. Results and Discussion

### 3.1. Preparation of TTN-LCNC by the Nanoprecipitation Method

Primary studies were carried out to carefully select the most proper method for the preparation of TTN-LCN. The nanoprecipitation method was used in which the TTN can be loaded during NC formation by evaporating the organic solvent. Solvent removal parameters such as evaporation temperature, time, and speed were selected based on the characteristics of the prepared nanocapsules (such as size and PDI) and previous studies [12, 13, 31]. In general, low-boiling solvents such as acetone with Δ*H*_vap_ of 32 kJ mol^−1^ or less could be completely removed under reduced pressure. Since acetone does not form any azeotrope with water and considering a part of the water was eliminated by evaporation at 40°C under reduced pressure, it can be concluded that acetone was completely removed from the water-acetone mixture [32]. Furthermore, the incomplete removal of organic solvent from the system makes the system physically unstable due to the Ostwald ripening phenomena and the particle size of the prepared nanoparticles will be increased [33]. So the reported characteristics of the nanocapsules such as size and PDI are another reason to indicate the proper selection of the solvent removal condition considerably. Different surfactants were tested, and Span 80 was chosen as it produced the minimum particle size. Also, several oils as lipid-core NCs were tested, and MCT oil was chosen as it improved EE% and particle size. The prepared NCs with MCT oil and Span 80 had a particle size range of 110–230 nm. So, a limitation was applied on size to prepare the smallest NC during preparation optimization based on the obtained size range in the primary works. This was done using Design-Expert® software to afford the formulation with maximum EE% and minimum size for dermal administration.

### 3.2. TTN-LCNC Characterization

#### 3.2.1. Characterization of the NCs

The particle size, PDI, zeta potential, and EE% of the NCs (15 runs) are exhibited in Table 3.  Table 4 reports the physicochemical properties of the optimized formulation.

#### 3.2.2. Effect of Independent Variables on EE%

**Figure 1:**
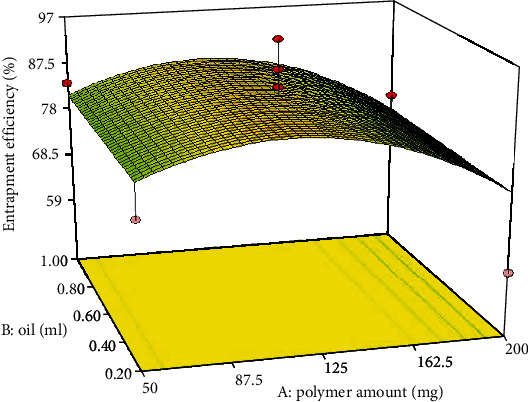
Response 3D plots for the effect of polymer amount (*A*) and oil amount (*B*) on entrapment efficiency (%).

The ability of NCs to encapsulate significant TTN amounts is necessary for the targeted topical treatment for acne. Values of the EE are reported in Table 3.  Figure 1 shows the response surface plot for the effects of the concentration of polymer amount (*A*) and drug amount (*B*) on the EE%. The ANOVA test for the observed EE% data indicates that the quadratic model was significant and fitting for the data. The final equation in terms of coded factors was as follows:

(3) $$\begin{array}{c} \text{EE}\left(\%\right)=+86.61-3.45\times A+16.66\times C-8.96\times A^{2}-12.77\times B^{2}. \end{array}$$

During the preparation of TTN-LCNC, the TTN was introduced in the organic phase. So, TTN will probably be entrapped in the inner lipid core of the nanocapsules (LCNC). The equation reveals that there was a significant effect (*p* = 0.0002) of the concentration of TTN on the EE%. The increased amount of encapsulated TTN with increasing concentration could be due to the saturation of the organic medium with TTN that forces the drug to be encapsulated into NCs [34]. Also, when comparing two formulations, considering that the same volume of the oil was entrapped inside the NCs, so the increased concentration of TTN in this medium will mean that more amount of the TTN will be entrapped inside the NCs. In addition, it was shown that by increasing the polymer amount from 110 mg, the TTN leakage could occur, and therefore, the EE% would decline [35].

#### 3.2.3. Effect of Independent Variables on Particle Size and PDI

**Figure 2:**
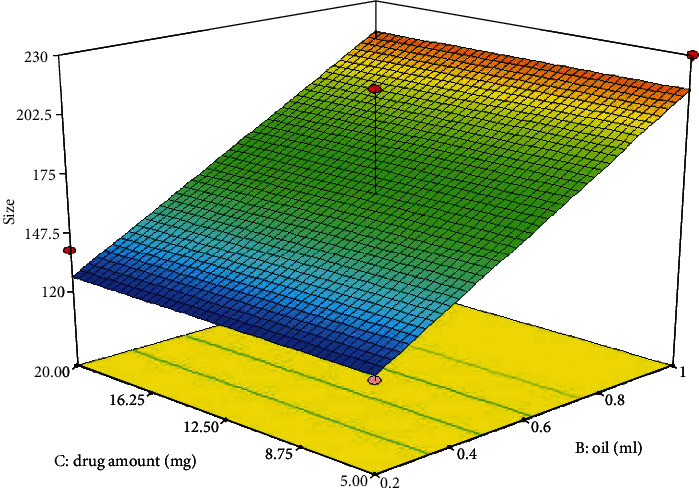
Response 3D plots for the effect of oil amount (*B*) and drug amount (*C*) on particle size.

The particle size of the TTN-LCNC is reported in Table 3. Also, Table 3 shows that the PDI of all TTN-LCNC was less than 0.4, indicating suitable homogeneity and narrow particle size distribution.  Figure 2 shows the response surface for the effects of the concentration of oil (*B*) and drug amount (*C*) on the particle size. The ANOVA test for the observed particle size data indicates that the linear model was significant and fitting for the data. The final equation in terms of coded factors was as follows:

(4) $$\begin{array}{c} \text{Size}=+171.00+43.75\times B. \end{array}$$

NCs with very small sizes (less than 600 nm) delivered their contents into deeper layers of the skin [36]. The positive coefficient of the term, *B*, indicates that the oil amount had a synergistic effect on the particle size of the prepared NCs (*p* = 0.0001). The ANOVA results revealed the significant effect of oil amount components on the particle size (*p* = 0.0001). This may be attributed to the LCNC. This may have resulted in the increased diameter of the lipid core, and hence, particle size increased.

#### 3.2.4. Effect of Independent Variables on Zeta Potential

Zeta potential is the measure of the overall charges of NCs and can be used to evaluate the stability of colloidal dispersions. When the zeta potentials of the nanoparticles are more than +30 mV or less than –30 mV, they are considered a stable colloidal suspension system due to electrical repulsion between particles [37]. The values of zeta potential for the TTN-LCNC are reported in Table 3.

### 3.3. Formulation Optimization and Analysis

In our strategy, a ratio greater than 4 (the desirable value) was observed in both responses, as shown in Table 4. The predicted *R*^2^ was calculated as a measure of how well the model predicts a response value [38]. The adjusted *R*^2^ should be within approximately 0.20 of the predicted *R*^2^ to be in reasonable agreement [39]. Otherwise, the data or the model might be a problem. Also, the predicted *R*^2^ values and adjusted *R*^2^ were in a reasonable agreement in both responses (Table 4). Then, the Design-Expert® software suggested an optimized formulation with overall desirability of >0.860 (Table 5). Therefore, the optimized formulation (F16) was selected for further studies. The selected formulation had a polymer amount of 110.47 mg, TTN amount of 17.39 mg, and oil amount of 0.2 ml. Solution number 1 was prepared and evaluated, and the residual between the predicted and observed responses was small, demonstrating the validity of the optimization process.  Table 6 reports the physicochemical properties of the optimized formulation.

### 3.4. Stability of the TTN-LCNC after UV Radiation

**Figure 3:**
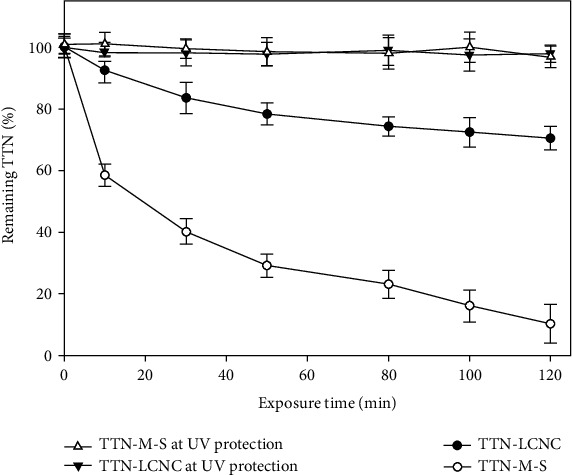
Photo-degradation of tretinoin-loaded lipid-core nanocapsules (TTN-LCNC) and TTN methanolic solution (TTN-M-S) of time (h). Results are shown as mean ± SD (*n* = 3).

The TTN-LCNC degraded 29.49% ± 3.90 after 2 h of exposure to UV radiation, and the TTN-M-S degraded 89.75 ± 4.34% after the same exposure time (Figure 3). As it is obvious, the preparation of nanocapsules can enhance the photostability of TTN upon exposure to UV radiation by approximately three times (*p* < 0.05). This means that the polymeric matrix could significantly screen out the UV radiation from the degradation of the TTN-LCNC.

### 3.5. Morphological Study

**Figure 4:**
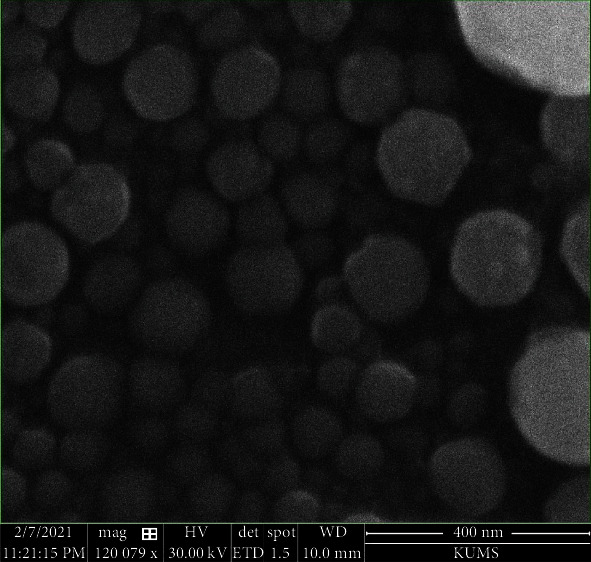
Representative SEM photographs of the selected tretinoin-loaded lipid-core nanocapsules.

The SEM-based photographs revealed spherical polymeric selected TTN-LCNC (Figure 4), and from these photographs, the dry NC diameters were evaluated and matched to the mean diameters measured by photon correlation spectroscopy.

### 3.6. Physical Stability

The optimized formulation exhibited high stability in size and EE% with no signs of aggregation during the 90 days of storage at 25°C and 4°C temperatures (Table 7).

### 3.7. TTN Release from NCs

**Figure 5:**
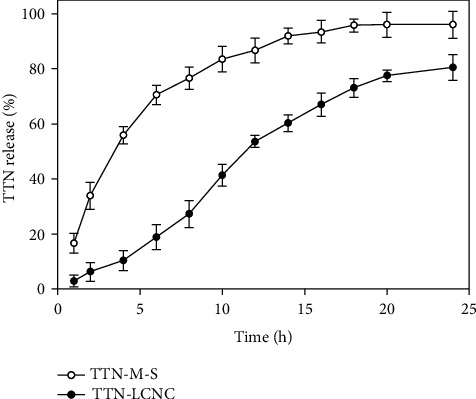
Release of the tretinoin-loaded lipid-core nanocapsules (TTN-LCNC) and TTN methanolic solution (TTN-M-S). Results are shown as mean ± SD (*n* = 3).

The *in vitro* TTN release from the NC suspensions was investigated over 24 h. The results are reported in Figure 5. In the early hours of the *in vitro* study, the TTN release from NCs was less than 10%, probably because of the slow release of TTN from NCs, while in the designated time period, more than 30% of TTN was released from TTN methanolic solution (i.e., TTN-M-S). As shown in the figure, in both formulations, the release of TTN was increased through the 24-hour incubation time and the final cumulative percent of TTN which was released from the NCs after 24 h was 80.5 ± 4.59% while the final cumulative release percentage of TTN from TTN-M-S was reported as 90 ± 3.64. In other words, the TTN release from NCs was significantly slower than the TTN-M-S after 24 h (*p* < 0.05). So, due to this release method, the prepared NCs are able to release the incorporated drug in a sustained manner.

### 3.8. Characterization of HG-TTN-LCNC

Table 8 reports the physicochemical properties of semisolid formulations containing TTN for dermatological administration compared to the TTN-LCNC. The presence of TTN changes the color of the gels to slightly yellow. Also, TTN did not change the pH range of the formulations. The drug content of formulations containing TTN was near to the expected values of 0.50 mg/g.

### 3.9. Skin Permeation/Retention Studies

**Figure 6:**
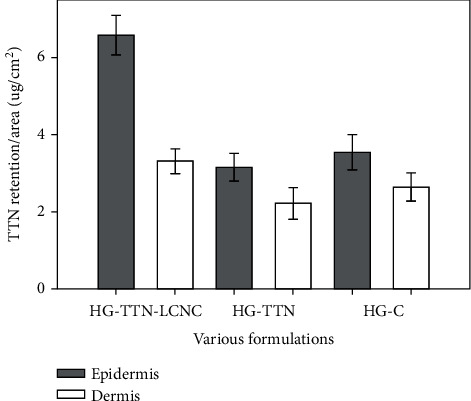
Skin retention (*μ*g/cm^2^) of hydrogel containing tretinoin-loaded lipid-core nanocapsules (HG-TTN-LCNC), tretinoin solution (HG-TTN), and commercial gel (HG-C) in the abdominal rat skin. Results are shown as mean ± SD (*n* = 3). All formulation was 0/025% *w*/*w*.

First, the ability of HG-TTN-LCNC to deliver TTN to the rat skin was evaluated and compared with the HG-TTN and HG-C. The retention results are provided in Figure 6. HG-TTN-LCNC could deliver significantly higher amounts of TTN to the epidermis layer compared to HG-TTN and HG-C (*p* < 0.05). Drug delivery to the dermis layer was minimal, and TTN was not detected in the receptor compartment. This outcome was expected due to the high octanol/water partition coefficient of TTN [40]. However, the slightly enhanced permeation capabilities of HG-C can be attributed to the skin penetration enhancement in their formulations [41]. Our work presents an enhancement of TTN deposition in the uppermost layer of the skin by its encapsulation in LCNC.

### 3.10. Irritation Test

**Figure 7:**
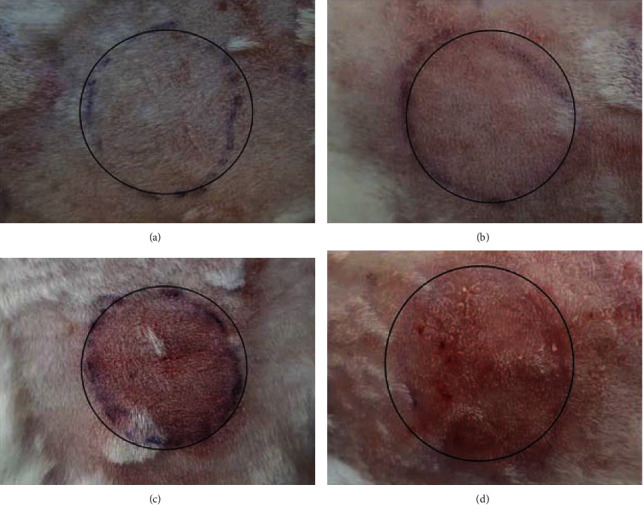
Photographs of the skin irritation study in the singled group at 72 h after administration of hydrogel containing the following: (a) tretinoin-loaded lipid-core nanocapsules (TTN-LCNC); (b) blank lipid-core nanocapsules (HG-B-LCNC); (c) tretinoin solution (TTN); (d) commercial gel. All formulation was 0/05% *w*/*w*.

One of the major side effects of the TTN is skin irritation (erythema), which strongly limits its utility and compliance by the patients. Ideally, the TTN delivery system should reduce or abolish these erythematic events. However, most of the marketed dosage forms (creams, lotions, and gels) are not able to reduce the irritation caused by TTN topical therapy [6]. The results obtained from the skin irritation studies are reported in Table 9, and the photographs are depicted in Figures 7(a)–7(d). The Draize test is a reliable method, and the results obtained from this study can be linked to those obtained from humans. This considerably less irritation could be associated with the small nanoparticle size and the role of NCs in protecting the skin tissue from direct contact with the TTN which was loaded in the NCs. Embedment of TTN in NCs would reduce the contact of the acidic group (COOH) of TTN with the stratum corneum and allow slow delivery of TTN to the dermis, hence reducing the irritation and increasing dermal tolerability [42]. The small nanoparticle size and the high entrapment efficiency of TTN in this selected formulation could reduce skin erythema. Thus, HG-TTN-LCNC is improving the skin tolerance of TTN and increasing patient compliance compared to marketed dosage forms.

## 4. Conclusion

In the current work, topical TTN-loaded cationic nanocapsules were investigated to enhance their pharmacodynamic efficacy, user compliance, and photostability. The Box-Behnken (BB) design was employed to statistically optimize the formulation variables. The optimum nanocapsule formulation (F16) displayed spherical morphology, reasonable drug EE%, and optimum particle size with cationic charge. Nanocapsules provided an increased photostability of tretinoin, as well as a prolonged drug release. The performed *in vivo* skin deposition studies suggested that nanocapsules, due to their cationic charge, promoted drug deposition into the epidermal region rather than the deep skin, compared to drug solution. Moreover, the optimized formulation of TTN-loaded nanocapsules showed skin tolerability by the skin irritation study. In conclusion, the results revealed the promising potential of cationic nanocapsules for topical delivery of tretinoin

## Figures and Tables

**(a):** 

Factors (independent variables)	Levels		
	High (+1)	Medium (0)	Low (-1)
*X*_1_: polymer amount (mg)	200	125	50
*X*_2_: drug amount (mg)	20	12.5	5
*X*_3_: oil amount (ml)	1	0.6	0.2

**(b):** 

Responses (dependent variables)	Constraints
*Y*_1_: encapsulation efficiency (%)	Maximize
*Y*_2_: particle size (nm)	Minimize

**Table 2:** Formations of the three-level three-factor design for the formulation of encapsulated TTN.

Run	Factor levels in actual values		
	*X*_1_: polymer amount (mg)	*X*_2_: drug amount (mg)	*X*_3_: oil amount (ml)
Midpoints			
1	200	20	0.6
2	50	12.5	1
3	200	12.5	1
4	50	20	0.6
5	200	5	0.6
7	125	5	1
9	200	12.5	0.2
10	125	20	0.2
11	50	5	0.6
13	50	12.5	0.2
14	125	20	1
15	125	5	0.2
Center points			
6	125	12.5	0.6
8	125	12.5	0.6
12	125	12.5	0.6

**Table 3:** Particle size, PDI, zeta potential, and EE% of encapsulated TTN (*n* = 3).

Formula	Particle size (nm ± SD)	PDI (values ± SD)	Zeta potential (mV ± SD)	EE (% ± SD)
1	151.7 ± 6.8	0.168 ± 0.059	43.7 ± 3.1	81.25 ± 6.57
2	230.2 ± 8.3	0.300 ± 0.082	44.7 ± 2.7	83.50 ± 7.17
3	201.7 ± 6.5	0.256 ± 0.065	34.3 ± 3.8	76.95 ± 8.36
4	171.4 ± 7.9	0.347 ± 0.058	32.3 ± 4.2	88.43 ± 6.73
5	167.5 ± 6.8	0.161 ± 0.052	38.8 ± 4.5	51.51 ± 4.55
6	162.3 ± 4.3	0.220 ± 0.074	26.2 ± 3.7	88.12 ± 9.74
7	230.8 ± 7.4	0.359 ± 0.063	33.9 ± 4.6	49.81 ± 7.62
8	157.2 ± 8.3	0.160 ± 0.074	34.7 ± 3.5	90.42 ± 4.77
9	115.9 ± 6.7	0.168 ± 0.056	26.1 ± 7.3	59.23 ± 5.38
10	140.1 ± 9.4	0.204 ± 0.070	38.4 ± 5.7	91.58 ± 4.35
11	178.6 ± 5.2	0.198 ± 0.042	34.9 ± 3.6	52.43 ± 5.48
12	215.9 ± 3.7	0.248 ± 0.065	29.2 ± 2.9	93.22 ± 2.37
13	127.4 ± 8.4	0.230 ± 0.049	28.1 ± 4.9	74.59 ± 8.61
14	196.7 ± 5.3	0.281 ± 0.080	31.4 ± 5.6	79.37 ± 9.38
15	125.6 ± 4.7	0.173 ± 0.059	30.1 ± 7.2	57.14 ± 4.21

**Table 4:** Results of regression analysis for responses *Y*_1_ (EE%) and *Y*_2_ (particle size).

	Model	*R* ^2^	Adjusted *R*^2^	Predicted *R*^2^	Adequate precision	SD	%CV	*p* value
*Y*_1_: EE%	Quadratic	0.8202	0.7483	0.6050	9.514	8.33	11.10	0.001
*Y*_2_: PS	Linear	0.7928	0.7769	0.7360	13.658	17.54	10.26	<0.0001

**Table 5:** The suggested formulations by Design Expert® 14.0.0 software.

Solution number efficiency	Polymer amount (mg)	Oil amount (ml)	Drug amount (mg)	Size (nm)	Entrapment	Desirability
1	110.47	0.2	17.39	127.25	92.3736	0.898^∗^
2	113.86	0.2	17.80	127.25	92.3182	0.897
3	103.97	0.2	17.30	127.25	92.3027	0.897
4	105.54	0.2	18.92	127.25	91.8058	0.892
5	99.81	0.2	15.61	127.25	91.4696	0.888
6	84.98	0.2	16.10	127.25	90.9541	0.883
7	87.33	0.2	20.00	127.25	89.9737	0.872
8	74.11	0.2	14.97	127.25	88.9248	0.861
9	65.45	0.2	18.52	127.25	88.8467	0.860

**(a):** 

Factor	Optimized level
*X*_1_: polymer amount (mg)	110.47
*X*_2_: oil amount (ml)	0.2
*X*_3_: drug amount (mg)	17.39

**(b):** 

Response (%)^a^	Expected	Observed error	Prediction
*Y*_1_: EE (%)	92.37	83.20 ± 3.27	-11.02
*Y*_2_: particle size (nm)	127.25	116.3 ± 5.6	-9.40

Prediction Error(%) = ((Observed‐Expected)/Observed) × 100.

**Table 7:** Size, PDI, zeta potential, and EE% of encapsulated TTN as a function of storage time. Results are shown as mean ± SD (*n* = 3).

Temperature	Day of storage	Size (nm)	PDI	Zeta potential (mV)	EE (%)
4°C temperature	0	114.9 ± 5.4	0.14 ± 0.021	32.5 ± 4.5	80.21 ± 2.38
	30	118.5 ± 5.8	0.18 ± 0.031	33.2 ± 3.6	79.72 ± 2.62
	60	115.2 ± 7.2	0.19 ± 0.037	34.6 ± 3.8	77.87 ± 3.43
	90	122.6 ± 7.5	0.20 ± 0.035	34.7 ± 4.3	77.16 ± 3.77
Room temperature	0	115.7 ± 6.7	0.16 ± 0.034	32.3 ± 2.7	81.82 ± 2.25
	30	118.5 ± 7.1	0.17 ± 0.032	34.6 ± 3.4	77.55 ± 3.19
	60	123.8 ± 7.4	0.20 ± 0.028	33.8 ± 4.1	76.78 ± 3.73
	90	128.4 ± 7.3	0.19 ± 0.034	34.6 ± 3.9	75.43 ± 3.43

**Table 8:** Physicochemical properties of the encapsulated TTN (TTN-LCNC), blank gel (HG-B), nonencapsulated TTN gel (HG-TTN), nanocapsule non-TTN gel (HG-LCNC), and encapsulated TTN gel (HG-TTN-LCNC). Results are shown as mean ± SD (*n* = 3).

Formulation	pH	Drug content (mg/g)	Viscosity (cP)	Aspect
TTN-LCNC	5.52 ± 0.10	0.501 ± 0.02	3.68 ± 0.10	Opalescent, yellow
HG-B	6.46 ± 0.12	—	7809.9 ± 152.91	Transparent, colorless
HG-TTN	6.53 ± 0.18	0.498 ± 0.03	7820.76 ± 119.67	Transparent, yellow
HG-LCNC	6.65 ± 0.15	—	7798.03 ± 174.19	Opalescent, white
HG-TTN-LCNC	6.62 ± 0.13	0.495 ± 0.02	7830.7 ± 102.26	Opalescent, slightly yellow

**Table 9:** Mean erythemal scores observed for encapsulated TTN gel (HG-TTN-LCNC), nanocapsule non-TTN gel (HG-LCNC), nonencapsulated TTN gel (HG-TTN), and commercial gel (HG-C) obtained at the end of 24, 48, and 72 h.

Formulation	Erythemal scores (*n* = 4)		
	24 h	48 h	72 h
HG-TTN-LCNC	0	0	0
HG-LCNC	0	0	0
HG-TTN	1	1	1
HG-C	1	2	2

## Data Availability

Data are available on request.
